# Sexual Selection of Human Cooperative Behaviour: An Experimental Study in Rural Senegal

**DOI:** 10.1371/journal.pone.0044403

**Published:** 2012-09-12

**Authors:** Arnaud Tognetti, Claire Berticat, Michel Raymond, Charlotte Faurie

**Affiliations:** 1 University of Montpellier 2, Montpellier, France; 2 Institute of Evolutionary Sciences, Centre National de la Recherche Scientifique, Montpellier, France; Durham University, United Kingdom

## Abstract

Human cooperation in large groups and between non-kin individuals remains a Darwinian puzzle. Investigations into whether and how sexual selection is involved in the evolution of cooperation represent a new and important research direction. Here, 69 groups of four men or four women recruited from a rural population in Senegal played a sequential public-good game in the presence of out-group observers, either of the same sex or of the opposite sex. At the end of the game, participants could donate part of their gain to the village school in the presence of the same observers. Both contributions to the public good and donations to the school, which reflect different components of cooperativeness, were influenced by the sex of the observers. The results suggest that in this non-Western population, sexual selection acts mainly on men’s cooperative behaviour with non-kin, whereas women’s cooperativeness is mainly influenced by nonsexual social selection.

## Introduction

Cooperative behaviour provides a benefit to the recipient at the expense of the actor and can therefore only be selected for if it also provides benefits to the actor who suffered the costs [1], [2]. Therefore, cooperation can evolve and spread in a population provided that it entails either direct or indirect benefits to the actors. In animals, cooperative behaviour is almost exclusively restricted to kin groups, apart from rare and specific cases of repeated encounters between pairs of individuals [3]. In humans, individuals cooperate in large groups involving non-relatives, in situations where no direct reciprocity is possible. Understanding the evolution of human cooperation in large groups and between unrelated individuals thus remains a challenging problem for economists [3] and evolutionary biologists [4], [5].

Theoretical and experimental studies show that the reputation acquired by cooperators is salient for future social interactions because it allows them to obtain future reciprocating partners [6], [7], [8], [9], [10]. For example, among the Ache in Paraguay, those hunters who share more also receive more food during hard times [11]. If cooperative individuals are most often chosen as partners due to the benefits they confer, then competition to be more cooperative than others can result (*competitive altruism hypothesis* [12], [13], [14]). In other words, individuals may behave altruistically for reasons related to their reputation because selective benefits (associated with status) accrue to the generous [15]. These benefits may include increased reproductive success.

Social selection, i.e. selection occurring as a consequence of interactions among individuals of the same species, is thus an evolutionary force that could potentially explain cooperation between non-kin individuals [16], [17], [18]. This is supported by several experimental studies using economic games. For example, in dyadic games, people actively compete to be more generous than others when they can benefit from being chosen for cooperative partnerships, and generous people are chosen more often as cooperative partners [19], [20]. Moreover, in a public good game followed by an opportunity to select a partner for a second game, cooperators are more often selected as partners [21]. Social selection thus appears to be a relevant evolutionary explanation of human cooperation in large groups of unrelated individuals, but this concept has induced surprisingly little empirical research [15], [22], [23].

A particular case of social selection is sexual selection: competition for access to mates could induce positive selection for cooperative behaviour. Some observations in animal species indeed indicate that sexual selection could be implicated in maintaining cooperation. In lance-tailed manakins (*Chiroxiphia lanceolata*), cooperative efforts are made by male pairs to engage in duet songs and dances for the purpose of mating with females [24], [25], [26]. In chimpanzees (*Pan troglodytes*), males exchange political support for mating opportunities [27]: the dominant male selectively tolerates males who have supported him most frequently in conflicts, so they copulate more often than other males (independent of their rank in the hierarchy).

In humans, there is some evidence that cooperative individuals are more attractive. In one study in a Western country, participants were recruited to play a series of dyadic economic games with a series of partners presented as facial photographs. They had to rate the attractiveness of their partners before and after each game. The results indicated that cooperating increased one’s attractiveness [28]. Moreover, self-reports suggested that cooperative traits are implicated in mate choice and that the preference for cooperative individuals is more pronounced in women [29]. Finally, a study showed that men (and not women) made more generous donations when they were observed by an individual of the opposite sex than by a same-sex individual or when nobody observed them [30]. The social reputation and prestige acquired through cooperative behaviour could thereby play a major role in mate choice. Moreover, cooperativeness appears to be preferentially utilised by men as a display to attract mates.

We therefore suggest that at least in some situations, cooperation involving non-kin individuals evolved partly due to increased access to mates. Several studies have shown that the presence of women elevates testosterone levels [31], [32] and physical risk taking in men [33]. Altruistic acts represent costly and risky behaviour, and they could therefore be used to indicate a potential mate’s qualities (*theory of costly signalling* [34], [35]), which may be transmitted to offspring [28], [36], [37]. For example, Meriam hunters who share costly turtle meat exhibit an earlier onset of reproduction, gain more mates of higher quality, and have more co-resident sexual partners than other men [38], [39]. Alternatively, cooperative behaviour could be used by potential mates as a proxy for potential future parental investment. Indeed, parental investment is a form of cooperative behaviour (implying a cost for the cooperator as well as benefits in the form of improved offspring fitness). This hypothesis implies a positive link between cooperativeness with non-kin individuals and parental investment, but no study performed thus far has investigated this link. In humans, investment is biparental; thus, both sexes might be sensitive to the cooperativeness of potential mates.

Surprisingly, the hypothesis that sexual selection could shape human cooperation has not received much attention in the recent literature, perhaps due to the difficulty of investigating this question [15], [23]. In addition, to our knowledge, no study has tested whether there is an effect of sexual selection using economic games in a non-Western culture. As human populations vary substantially in their level of individual cooperation [40], [41], the potential influence of sexual selection for cooperative behaviour could be culture-dependent. The aim of our study was therefore to test whether there are effects of sexual selection on cooperativeness and to possibly extend previous conclusions to another culture, rural Senegal in this case.

We used two measures of cooperativeness. The first is a public good game (PGG), which is more similar to natural situations (such as sharing of food, collective hunting, and collective building) than dyadic games (used in [28]) or self-reports (used in [29]). A previous study showed that the presence of an audience increases contributions to a public good, but a potential sex effect was not investigated [42], and a study using a different game suggested that male and female players could react differently to different types of audiences [43]. Groups of four men or four women who were not direct kin were recruited to play in the presence of non-familiar observers, either of the same sex or of the opposite sex. Because several studies showed that males could have evolved motivations to display cooperative behaviour to coalitionary partners (e.g. [31]), out-group observers were used to prevent such potential effects that could confound the influence of sexual selection.

Our second measure of cooperative behaviour is a more naturalistic evaluation that involves a charitable contribution aimed at children, in a real-world context. At the end of the game, participants could donate part of their gain from the game to the village school in the presence of the same observers. As well as contributions to a public good, generosity is a costly behaviour, and therefore it can be used by men to signal their mate quality. Our two measures may reflect different components of cooperative behaviour. Indeed, although the PGG is widely used in experimental studies, mainly in Western societies but also in non-Western societies [41], [44], [45], studies examining the relationship between behaviour in a PGG and in real life are scarce [45], [46], [47], [48]. Our donation test is likely to be a more realistic situation than the PGG. Indeed, participants were not informed about the fact that they could donate part of their payoff until the end of the game. It was the local observers who suggested making a donation, as opposed to the rules of the game that were given by the experimenters at the beginning of the experiment. Moreover, charity is common in this population and the donation test does not involve complicated rules such as those of the PGG, where calculations are necessary. Finally, because these donations were for children, they are directly relevant in the context of our hypothesis that cooperative behaviour may be used as a signal of potential parental investment (for an indirect indication see the discussion section).

Because cooperativeness seems preferentially used by men as a mating effort [28], [29], [30], [38], [39], and because paternal investment shows larger inter-individual variations than maternal investment [49], [50], we predicted that cooperativeness as a mate choice criterion would be most relevant for women, such that male behaviour should be more influenced by the presence of opposite-sex observers than female behaviour.

## Methods

The protocols used to recruit participants and to collect data were approved by the French National Commission on Informatics and Liberties (CNIL) and the Senegalese National Council on Ethics in Health Research (CNERS). Written informed consent was obtained from all subjects or from a parent if subject was younger than 18 (n = 3).

### Study Population

The study was conducted in five rural villages located in the Sine Saloum area of Senegal, close to the west coast of Africa. Villages are distributed around a small city of approximately 12,000 inhabitants, and there are approximately 300 individuals in each village. The average distance between the five villages is 8 km. The mode of subsistence is mainly agricultural and is based on subsistence crops, such as millet as well as on cash crops, such as peanuts, cashew nuts, and sorrel. In the present study, the proportion of farmers exceeded 60%. The social system in the area is patrilocal, and the inheritance mode is patrilineal. Polygynous marriages are common, with a maximum of four wives, as permitted by the local interpretation of Islam, which most people practice. Generally, women are in charge of all household chores (e.g., buying food, cooking, cleaning, fetching water, taking care of children, gardening, and collecting wood), whereas men are in charge of fishing, working in the fields, and building houses. Most of the important decisions at the house and the village levels are made by the men.

### Demographic Data

A total of 39 groups of men and 30 groups of women, aged 16–78 years (mean ± s.d.: 39±16 for men and 38±14 for women), were recruited from the five villages, which were chosen because they each contained a school. The participants were recruited on a voluntary basis. Information was collected on their age, marital status, number of offspring, birth order, and socio-economic status. Socio-economic status was estimated by recording the land area, number of cattle, number and type of vehicles, and houses, and all of these factors were weighted by their average cost.

### Protocol

In each session, four men or four women were recruited to participate in a PGG. To guarantee experimenter-subject anonymity, a subject number was assigned to each participant. No detailed information about the aim of the experiment was given to the participants, and they were not told that the game would be followed by an invitation to make a donation aimed at children. At the beginning of each session, the instructions for the game were explained by a local research assistant in the native language of the participants. Decision-making took place inside a van, so that group members and other villagers could not observe the participants: only one participant was inside the van at a time, and only the observers, who stayed inside the van throughout the experiment, could see the participant’s decision. While one of the group members was in the van, the three others, outside the van, could see each other, but they were asked not to converse. To limit communication between the participants, the two experimenters isolated them at a private location to collect other information (height, weight, SES, and so on); most of the time, a player was either in the van with the observers, or outside with one of the experimenters, or alone.

The observers were recruited from a distant village, so that they would not be familiar to the players. The observers were presented to the participants as research assistants. To test the hypothesis that cooperation between same-sex partners in the game and generosity towards children are influenced by sexual selection, three groups of local observers were constituted. In the first condition, the participants and observers were of the opposite sex. In a control condition, the observers were of the same sex as the participants. We added a second control condition only for male participants in which the observers were post-reproductive women (over 50 years of age). Because old men are also potential mates in this population, even for young women, no control condition involving old men was implemented. To maximise the probability that at least some of the observers would be considered attractive by opposite-sex participants, (i) three observers were recruited in each category (men, young women, and old women); (ii) the three male observers were of different ages (approximately 25, 35, and 45 years of age); and (iii) the young female observers were not married. The attractiveness of the male observers and young female observers was rated by female and male players, respectively, at the end of the experiment on a scale of 1 (low attractiveness) to 4 (high attractiveness). The average attractiveness (mean ± s.d.) was 2.91±0.56 for male observers as rated by female players and 2.92±0.48 for young female observers as rated by male players.

### The Public Good Game

A PGG session included five periods (sequential game); the repetitions allowed the players to modify their strategies depending on how other group members played, and thereby including aspects of reciprocity in our evaluation of cooperative behaviour. In each period, each player received 200 g of rice. We used rice instead of, e.g., coins to obtain continuous rather than incremental data and because most participants were illiterate, which would have made calculations and conversions problematic. Moreover, rice is consumed daily in most families, and it is shared on a common plate during family meals. Food sharing is extremely important in the Senegalese culture. Finally, rice is particularly valued, as its taste is often considered to be better than any other cereal. Rice imported from Asia is preferred and is more expensive. When the prices dramatically increased in 2008, it became scarcely affordable for many families and therefore even more valued.

The participants had to decide how to allocate their initial endowment of rice to an individual good and a collective good. To this end, during each period, the players entered the van individually, where the observers were already present. Once inside, they allocated the totality of their endowment in front of the observers between their private good and the public good, which were represented by opaque boxes to ensure the anonymity of their allocations among the other players. Each player was given a tag of the same colour as his private box. The player then left the van, and another member of his group came in for the same task. At the end of each period, the players were informed about the total amount of rice invested by the group in the public good during this period. The players knew that the game would end after five periods.

The total amount of rice allocated to the public good was doubled at the end of the game and then divided into four equal gains for the four players, to which the amount of rice in their private good was added.

The payoff for each player was therefore Rice _private good_+(∑Rice _public good_×2)/4.

The payoffs were placed in opaque bags to ensure the secrecy of the amount won and given to each player by the observers in private.

### The Donation Test

When a participant received his/her final payoff in the van, he/she was informed by the observers (“assistants”) about the possibility of donating part of it to the school canteen of the village. It was specified that this donation was optional and that any amount of rice would be accepted. This donation represents a generous behaviour toward children, which is presumably a proxy for potential parental investment.

At the end of the game session, each player left the van with an opaque bag containing the payoff won during the public-good game minus the donation to children, if any.

### Data Collection

At the end of each period of the PGG, the observers secretly weighed the private rice fund of each player and the public fund of the group. They also weighed the amount of rice donated to the school canteen at the end of the experiment. The accuracy of the scale used was 1 g.

At the beginning and end of the experiment, when the observers and participants were of opposite sexes, the observers were asked to rate the attractiveness of each participant on a scale of 1 (low attractiveness) to 4 (high attractiveness).

### Statistical Analysis

#### Effect of the categories of observers on the contribution to the public good

To assess the potential influence of the different categories of observers on the players’ contribution to the public good, we used censored regression models (Tobit models) for male and female players separately (censReg function of the censReg package in R). The response variable was the amount of rice invested in the public good, left-bounded at 0 and right-bounded at 200 (with 200 g being the amount of rice provided at the beginning of each period). Two models were used for each sex: one in which the response variable was the amount of rice invested in the public good in the first period, representing the spontaneous contribution to the public good (before knowing how other members of the group played), and one for the second through fifth periods, which was a censored regression model for panel data, to account for the fact that an individual contribution in a period *t* would be influenced by the group’s contribution (total amount of rice invested by the group) in period *t-1*.

The explanatory variable tested was the category of observers (men, young women, and, for male players, old women). In addition, we controlled for the potential confounding effects of the player’s age, socio-economic status, and number of offspring, as well as the village where the experiment took place. We also controlled for the player’s birth order (firstborn or not) because firstborns were less trustful and reciprocated less than later-born and only children in a previous study [51]. Because age and offspring number were correlated (Spearman’s correlation test, ρ = 0.69, *p*<0.001), separate models were built with each of these two variables. The significance of the terms was evaluated using χ^2^ tests.

#### Effect of the observers’ sex on generosity towards children in the donation test

To investigate potential variations in the probability of donating to the school canteen as a function of the observers’ category, general linear mixed models (GLMMs) were used for each sex (lmer function of the lme4 package in R), allowing us to account for the random effect of the group. Due to the high proportion of individuals who did not make any donation (104 out of 276 participants), we used a binary response variable (0 for no donation, 1 for any non-zero donation). The explanatory variable tested was the category of observer (men, young women, and, for male players, old women). For male players, differences among the three categories of observers were also contrasted. The players’ age or number of offspring, socio-economic status, birth order, village, and payoff in the game were used as fixed effects. The significance of the terms was evaluated using χ^2^ tests.

To investigate the potential effect of the observers’ category on the amount of rice donated, linear mixed models (lme function of the nlme package in R) were applied for those participants who made a non-zero donation, fitted with a Gaussian error structure, with the same explanatory and control variables as in the previous models. For male participants, a log transformation was necessary to normalise the residuals. The significance of the terms was evaluated using *F* tests. Finally, for male players, differences among the three categories of observers were contrasted (glht function of the multcomp package in R).

#### Link between contributing to the public good and generosity in the donation test

To investigate whether cooperativeness in the PGG was linked to cooperativeness in the donation test, we used Spearman non-parametric correlation tests (cor.test function). Four tests were used for each sex: for the PGG, we either used the first period or averaged the second to fifth periods, and for the donation test, we either used the probability of donating or the amount of rice donated for those participants who made a non-zero donation.

#### Participants’ attractiveness as rated by the observers

To investigate whether cooperativeness influenced attractiveness as rated by the opposite-sex observers, we analysed the relationship between the change in perceived attractiveness (after *versus* before the tests) and either the players’ contribution to the public good (averaged across all periods) or their charitable contribution towards children (amount of rice donated). Linear regression models (lm function in R) were used for male and female players separately. The response variable was the difference between attractiveness when rated after the experiment and attractiveness when rated at the beginning of the experiment.

In all models, the player’s age or number of offspring, socio-economic status and village were used as control variables. In donation models, the payoff at the end of the PGG was also controlled for. In each analysis, a full model was built and was not subjected to stepwise simplification, which could have increased the number of false positives and, thus, provided non-conservative results [52]. The significance of the terms was evaluated using *F* tests. Additionally, the normality of the residuals and the homoscedasticity of the models were checked when necessary. All statistical analyses were performed using R software, version 2.10.1 [53].

## Results

### Effect of the Category of Observer on the Contribution to the Public Good

**Figure 1:**
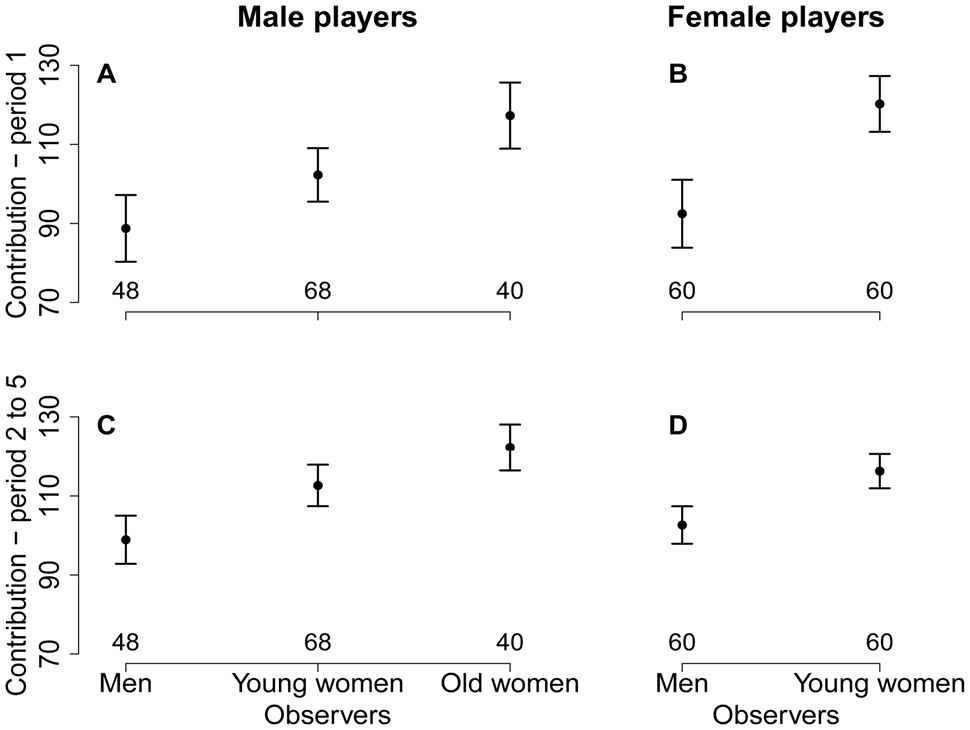
The influence of the categories of observers on the contribution to the public good, measured as the average amount of rice invested in the first period by (a) male and (b) female players and during the subsequent periods (2 through 5) by (c) male and (d) female players (raw data). With respect to men’s contributions, censored regression models did not show a significant influence of the category of observer, either during the first period of the PGG or during the subsequent periods. In contrast, the models indicated that women contributed more in the presence of young female observers than in the presence of male observers, during both the first period and the subsequent periods.

**Figure 2:**
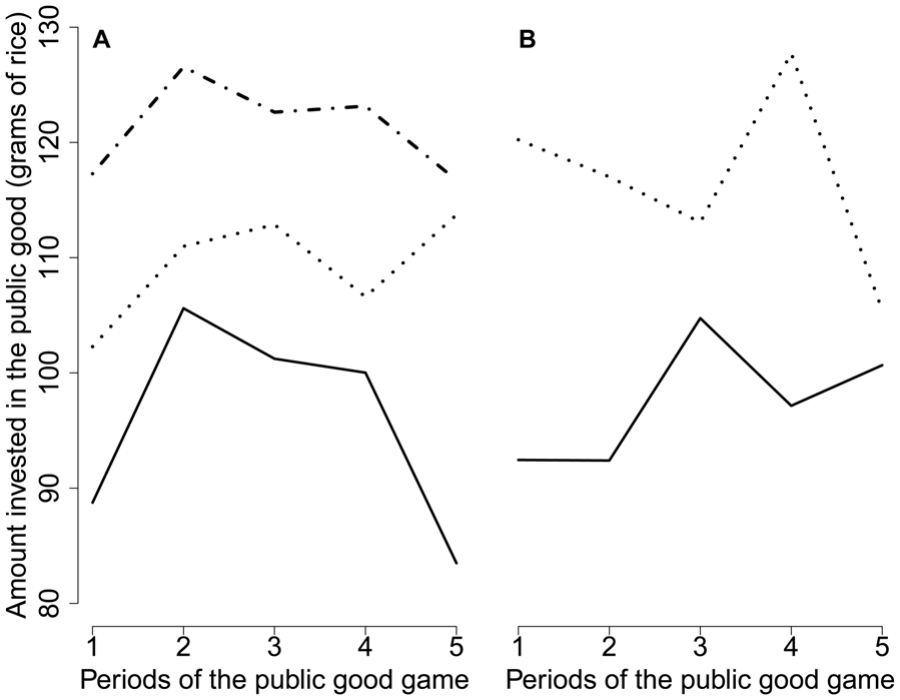
Contribution to the public good in each period of the PGG, made by (a) male participants and (b) female participants in presence of male observers (solid lines), young female observers (dotted lines) or old female observers (dotted-dashed lines). Men’s contributions tended to increase across periods in the presence of young female observers, reaching a maximum at the last period, although the typical decreasing pattern was observed for the two types of controls. Similarly, women’s contributions increased globally across the game periods in the presence of male observers and not in the presence of female observers.

The contribution to the public good by men was not significantly influenced by the category of observers during the first period of the game (χ^2^ = 3.2, *df* = 2, *p* = 0.21, see model in Table S1) or during the subsequent periods (χ^2^ = 5.1, *df* = 2, *p* = 0.08, see model in Table S2), despite the trends observed in the raw data (Figure 1). Women contributed significantly more in the presence of young female observers than in the presence of male observers during both the first period (χ^2^ = 7.2, *df* = 1, *p*<0.01, see model in Table S1) and the subsequent periods (χ^2^ = 9.5, *df* = 1, *p*<0.01, see model in Table S2), as was observable in the raw data (Figure 1). Figure 2 shows that men’s contributions to the public good tended to increase across the game periods in the presence of young female observers, reaching a maximum in the last period, although the decreasing pattern typically observed in a classical PGG [54], [55], [56], [57] was observed for the two types of controls. Similarly, women’s contributions increased globally across periods in the presence of male observers, but not in the presence of female observers.

### Effect of the Observers’ Sex on Generosity Towards Children in the Donation Test

**Figure 3:**
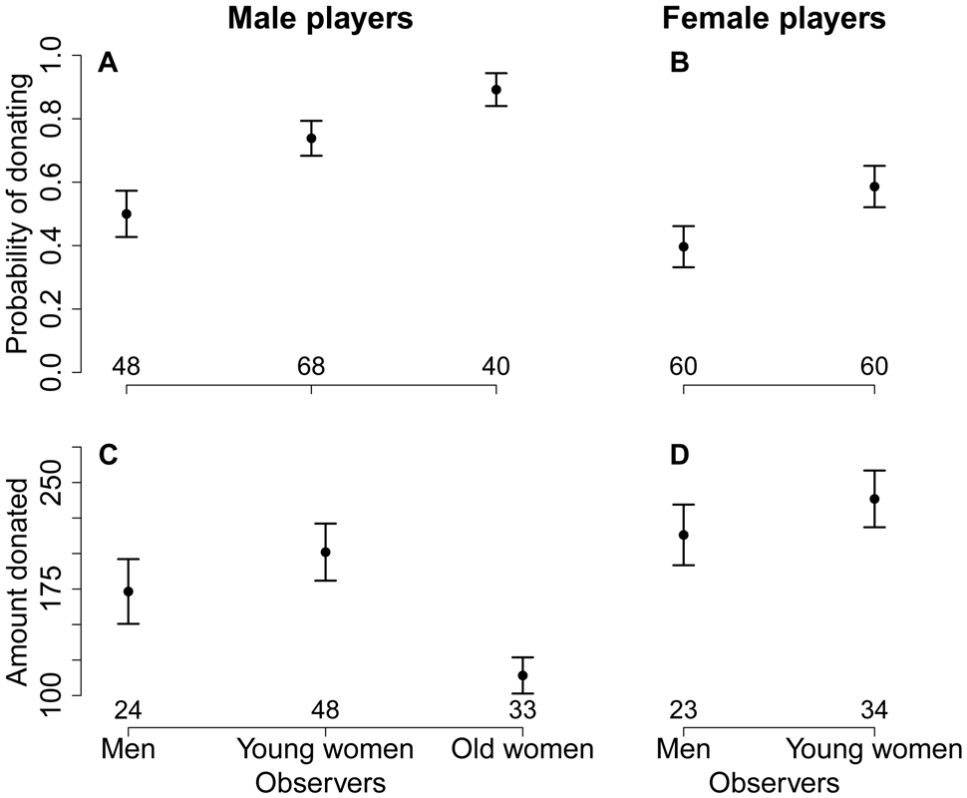
The influence of the categories of observers on generosity towards school children. Generosity was measured as the probability of donating among (a) male and (b) female participants, and as the amount donated by (c) male and (d) female participants who made a non-zero donation (raw data). Logistic regression models showed that men’s propensity to make a donation in the presence of young female observers was significantly higher than in the presence of male observers, but not significantly different from their probability of making a donation in the presence of old female observers. For the female participants, logistic regression models showed that they were more likely to donate in the presence of female observers than in the presence of male observers. Among the participants who made a non-zero donation, linear mixed models showed that men were more generous in the presence of young female observers than in the presence of old female observers, but not significantly more than in the presence of male observers. No significant difference was found in the amounts donated by women.

Men’s propensity to make a donation was significantly influenced by the category of observer present (χ^2^ = 12.9, *df* = 2, *p*<0.01, see model in Table S3). As observable in the raw data (Figure 3), post-hoc two-by-two contrasts showed that the male participants were more likely to give in the presence of young female observers than in the presence of male observers (χ^2^ = 7.5, *df* = 1, *p*<0.01), but not more than in the presence of old female observers (χ^2^ = 1.1, *df* = 1, *p* = 0.29). The amount donated was also significantly influenced by the category of observer (F_2,27_ = 4.5, *p* = 0.02, see model in Table S4): men were more generous in the presence of young female observers than in the presence of old female observers (*z* = 3.03, *p*<0.01), but not more than in the presence of male observers (*z* = 0.05, *p*>0.99) (Figure 3).

Women were more likely to make a donation in the presence of young female observers than in the presence of male observers (χ^2^ = 4.5, *df* = 1, *p* = 0.03, see model in Table S3), but no difference was found in the amount donated (*F*_1,15_ = 0.9, *df* = 1, *p* = 0.35, see model in Table S4) (Figure 3).

### Link between Contributing to the Public Good and Generosity in the Donation Test

We did not find a correlation between the contribution to the public good (either for the first period or for the subsequent periods) and charitable contribution (either for the probability to donate or for the amount donated) for either male or female players (ρ coefficients ranging from −0.18 to 0.01; *p*-values from 0.07 to 0.99).

### Participants’ Attractiveness as Rated by the Observers

We did not find any relationship between contributing to the public good or generosity towards children in the donation test and the change in the participants’ attractiveness following the tests (see model in Table S5 for men and S6 for women), whether controlling for attractiveness as rated before the game (*F* ranging from 0.001 to 3.01; *p*-values from 0.09 to 0.97) or not (*F* ranging from 0.01 to 2.87; *p*-values from 0.10 to 0.90).

## Discussion

Our results show that cooperativeness within groups of same-sex individuals and generosity towards children are uncorrelated components of cooperative behaviour and are both influenced by the sex of observers. However, the relative importance of sexual selection and nonsexual social selection appears to be different for men and women.

Although the observers’ sex did not influence men’s contribution to the public good, men’s charitable contribution increased in the presence of young women in the donation test. Charitable contributions could be used by men as a display to attract mates, and apparently, they do not represent the same component of cooperative behaviour as contributions to the public good. Although we designed the public-good game to make it as realistic as possible (rice was used instead of tokens, and the players were not anonymous to each other), the donation experiment is more similar to real-life situations. Indeed, this kind of behaviour is usual in this culture. Moreover, it does not involve complicated rules such as those of the PGG. Finally, as opposed to the PGG, this part of the experiment was presented by local observers and at the end of the experiment. It appears to be important that future studies use the production of real public goods as a measure of cooperativeness.

Nevertheless, men’s contribution tended to increase across the five periods of the PGG in the presence of female observers, although it decreased in the presence of male or old female observers (Figure 2), as is typically observed in a PGG [54]. This is potentially an indication that men’s contribution to the public good is also influenced by sexual selection. Indeed, men’s cooperative behaviour has previously been positively linked with their attractiveness to female raters [28]. We did not find a similar relationship between cooperativeness and attractiveness in our experiment. The small sample of attractiveness raters (three observers in each category) combined with the fact that field conditions were not optimal to focus on ratings, and not standardised (as in a lab), could partly explain this discrepancy.

Cooperative behaviour, such as charity donations in the present study, could be used by men to indicate a potential mate’s qualities, which may be transmitted to offspring [35]. Alternatively, it could also be used by potential mates as a proxy for future paternal investment. Indeed, as paternal investment is more variable than maternal investment, the inclination of women’s preferences towards cooperative men could be an adaptive strategy. Our study does not allow weighting the relative importance of these two explanations (which are not mutually exclusive). Nevertheless, in a previous study in the same Senegalese population, a high level of paternal investment was the second most cited trait (the first being a high level of men investment in his wife) by women who were asked to report the relevant characteristics when choosing a partner [58]. Besides, we have an indirect indication that generosity towards the school is linked to parental investment: after the experiment, observers had to evaluate the parental investment that each participant could potentially provide (“do you think that this person would be a good father/mother”). The results showed, for both male and female participants, that their generosity in the donation test was positively associated with their parental quality, as perceived by all categories of observers: male observers (male participants: F_1,38_ = 19.8, p<0.0001; female participants: F_1,35_ = 36.0, p<0.0001), young female observers (male participants: F_1,49_ = 27.4, p<0.0001; female participants: F_1,45_ = 21.7, p<0.0001) and old female observers (male participants: F_1,28_ = 4.2, p = 0.05). Although this evaluation was performed after the tests and therefore does not constitute a “guess”, these results suggest that generous individuals are likely to be viewed as good parents. However, only a proper empirical test could support this explanation.

As can be seen in Figures 1, 2 and 3 (left panels), men’s cooperativeness and probability of donating tended to be positively influenced by the presence of old female observers, but surprisingly, these effects were not significantly different from the effects associated with young female observers in any model, despite our large sample size (over 150 male participants). We included this second control in our experimental design (in addition to the first control, i.e., the same-sex condition) to further test the sexual selection hypothesis (assuming that post-reproductive women would not be regarded as potential mates by male participants). Our prediction was that men’s cooperativeness would be similar for both controls. Because the raw data exhibited different patterns between the two control treatments (male observers and old female observers), we compared them statistically. The results indicate that the presence of old female observers significantly increased men’s contributions to the public good during periods 2 through 5 (χ^2^ = 5.4, *df* = 1, *p* = 0.02) and their probability of donating (χ^2^ = 9.4, *df* = 1, *p*<0.01). This suggests that old women’s opinions were important to these men, most likely because old women play a key role in establishing an individual’s reputation in this population. Indeed, old women hold an important social position in these villages and strongly influence marriage decisions. In addition, men could be willing to improve their reputation as cooperators in the presence of women of all ages, perhaps because women are more likely to exchange information about men with each other. Future studies will investigate the influence of the interaction between sex and age of observers more precisely, including with old male observers.

Women contributed more to the public good and were more generous when observed by women than by men. Therefore, sexual selection does not seem to affect women’s cooperative behaviour. This could be because maternal investment is generally less variable than paternal investment, making cooperativeness a less relevant criterion when men choose a partner. Nonsexual social selection appears to have more influence on women’s cooperative behaviour in this population. Women seem to be more concerned with their reputation as cooperators among other women. Indeed, several observations made in the field indicate the importance of cooperation among women: within households, co-wives cooperate in child care, cooking, and chores; within neighbourhoods, they cooperate to fetch water from the well and work in the fields. They also gather in women’s councils to counterbalance men’s power, discuss common decisions and projects, resolve everyday problems, and improve their living conditions. Moreover, as women are the dispersing sex, they generally do not live in their natal village and cannot rely on their kin network. Cooperativeness with non-kin same-sex individuals therefore seems to be a vital component of women’s lives and livelihoods, and the results of our experiments are consistent with these observations.

Nevertheless, women’s contributions increased across the five periods of the public-good game in the presence of male observers (and not in the presence of female observers, see Figure 2). This pattern could potentially reflect involvement of sexual selection in women’s cooperative behaviour. In addition, in our study population, women who contributed more to the public good and who were more generous to the school canteen had more surviving offspring (see model in Table S1, S2 & S4). No effect of women’s socio-economic status was found in any of the examined models, which suggests that the observed link between cooperativeness/generosity and offspring number is not confounded by the level of resources. Cooperative women could have higher reproductive success through improved access to high quality mates and better offspring survival. Additionally, in this population, in which offspring from different co-wives are raised in the same household, choosing women who are cooperative toward non-kin could increase a man’s fitness. The positive relationship between women’s cooperativeness and their number of offspring could result from a positive influence of living in a globally cooperative household. Moreover, because intra-sex cooperation is crucial for the reputation and social status of women, choosing a cooperative woman could improve a man’s offspring status and access to resources at the household level. Further studies should examine whether cooperative women are indeed preferred by men as mates and whether the presence of cooperative co-wives is beneficial for children’s survival and success at the household level. Multi-level inter-group selection mechanisms [59] or ecological and demographic differences [45] could explain the considerable variations in cooperation levels observed between villages. This finding stresses the need to investigate the potential link between the average cooperativeness of a village’s inhabitants as measured by economic games and the efficiency of public goods management (e.g., of collective wells, mosques, infirmaries, fences, schools) in this village, controlling for relevant ecological variables (e.g., village wealth, social network size, frequency of market contact) and demographic variables (e.g., village population sizes or migration levels).

### Conclusions

Determining the relative importance of sexual selection and nonsexual social selection represents an empirical question [23], and to our knowledge, no study performed thus far has attempted to estimate the relative importance of these two mechanisms in selecting for cooperative behaviour. Our study population appears to be appropriate for distinguishing between these two mechanisms because the sex of social partners is different from the sex of sexual partners: cooperation during daily activities and tasks is mainly intra-sex, as indicated by our field observations. Our results show that the relative importance of sexual selection and nonsexual social selection is different for men and women: sexual selection appears to mainly influence men’s cooperativeness, whereas nonsexual social selection appears to be the main force for women. Nevertheless, these conclusions may be restricted to experiments with out-group observers such as in the present study: with in-group observers, male behaviour appears to be influenced by nonsexual social selection also [31].

Moreover, because human populations vary substantially in their levels of individual cooperation [40], [41], the relative importance of sexual and nonsexual social selection for cooperative behaviour could be culture dependent. Evaluating the relative importance of these two forms of selection in other cultures should provide significant insights into the evolution of human cooperation.

## Supporting Information

Table S1 Tobit regression models of (1) men’s and (2) women’s contributions to the public good in the first period of the PGG. For each variable, the estimate, standard error of the mean (SE), χ^2^ statistic, degrees of freedom (df), and *p*-value of the χ^2^ test are given. For categorical variables, the estimates are for one category compared to the reference category (underlined term). The results of the models controlling for age instead of the number of offspring were not qualitatively different (available upon request).
(PDF)

Table S2 Random Tobit regression models of (1) men’s and (2) women’s contributions to the public good in periods 2 through 5 of the PGG. For each variable, the estimate, standard error of the mean (SE), χ^2^ statistic, degrees of freedom (df), and *p*-value of the χ^2^ test are given. For categorical variables, the estimates are for one category compared to the reference category (underlined term). The results of the models controlling for age instead of the number of offspring were not qualitatively different (available upon request).
(PDF)

Table S3 GLMMs of (1) men’s and (2) women’s probability of donating to the school canteen. For each variable, the estimate, standard error of the mean (SE), χ^2^ statistic, degrees of freedom (df), and *p*-value of the likelihood ratio test of the comparison between the full model and the model without the factor, are given. For categorical variables, the estimates are for one category compared to the reference category (underlined term). The results of the models controlling for age instead of the number of offspring were not qualitatively different (available upon request).
(PDF)

Table S4 LMMs of the amounts donated by (1) men and (2) women to the school canteen for individuals who made a non-zero donation. For each variable, the estimate, standard error of the mean (SE), *F* statistic, numerator degrees of freedom (df), denominator degrees of freedom (dfden), and *p*-value of the likelihood ratio test of the comparison between the full model and the model without the factor, are given. For categorical variables, the estimates are for one category compared to the reference category (underlined term). The results of the models controlling for age instead of number of offspring were not qualitatively different (available upon request).
(PDF)

Table S5 General linear regression of the change in men’s attractiveness as rated by (1) young female and (2) old female observers as a function of the men’s (a) contributions to the public good and (b) generosity towards children. For each factor, the estimate, standard error of the mean (SE), degrees of freedom (df), *F* statistic, and *p*-value of the likelihood ratio test of the comparison between the full model and the model without the factor, are given. For categorical variables, the estimates are for one category compared to the reference category (underlined term).
(PDF)

Table S6 General linear regression of the change in women’s attractiveness as rated by male observers as a function of the women’s (a) contributions to the public good and (b) generosity towards children. For each factor, the estimate, standard error of the mean (SE), degrees of freedom (df), *F* statistic and *p*-value of the likelihood ratio test of the comparison between the full model and the model without the factor, are given. For categorical variables, the estimates are for one category compared to the reference category (underlined term).
(PDF)

## References

[1] Lehmann L, Keller L (2006) The evolution of cooperation and altruism. A general framework and classification of models. J Evol Biol 19: 1365–1378. 16910958 10.1111/j.1420-9101.2006.01119.x

[2] West SA, Griffin AS, Gardner A (2007) Evolutionary explanations for cooperation. Curr Biol 17: 661–672. 10.1016/j.cub.2007.06.004 17714660

[3] Fehr E, Fischbacher U (2003) The nature of human altruism. Nature 425: 785–791. 14574401 10.1038/nature02043

[4] Alexander RD (2006) The challenge of human social behavior. Evol Psychol 4: 1–28.

[5] Boyd R, Richerson PJ (2009) Culture and the evolution of human cooperation. Philos Trans R Soc Lond B Biol Sci 364: 3281–3288. 19805434 10.1098/rstb.2009.0134 PMC2781880

[6] Fehr E (2004) Don’t lose your reputation. Nature 432: 449–450. 15565133 10.1038/432449a

[7] King Casas B, Tomlin D, Anen C, Camerer CF, Quartz SR, et al (2005) Getting to know you: reputation and trust in a two-person economic exchange. Science 308: 78–83. 15802598 10.1126/science.1108062

[8] Nowak MA, Sigmund K (1998) Evolution of indirect reciprocity by image scoring. Nature 393: 573–577. 9634232 10.1038/31225

[9] Ostrom E (2003) Toward a behavioral theory linking trust, reciprocity, and reputation. In: Ostrom E, Walker J, editors. Trust and reciprocity: Interdisciplinary lessons from experimental research. New York: Russell Sage Foundation. 19–79.

[10] Wedekind C, Braithwaite VA (2002) The long-term benefits of human generosity in indirect reciprocity. Curr Biol 12: 1012–1015. 12123575 10.1016/s0960-9822(02)00890-4

[11] Gurven M, Allen Arave W, Hill K, Hurtado M (2000) “It’s a wonderful life”: signaling generosity among the Ache of Paraguay. Evol Hum Behav 21: 263–282. 10899478 10.1016/s1090-5138(00)00032-5

[12] Noe R, Hammerstein P (1995) Biological Markets. Trends Ecol Evol 10: 336–339. 21237061 10.1016/s0169-5347(00)89123-5

[13] Noe R, Hammerstein P (1994) Biological Markets - Supply-and-Demand Determine the Effect of Partner Choice in Cooperation, Mutualism and Mating. Behav Ecol Sociobiol 35: 1–11.

[14] Roberts G (1998) Competitive altruism: from reciprocity to the handicap principle. Proc R Soc Lond B Biol Sci 265: 427–431.

[15] Nesse RM (2007) Runaway social selection for displays of partner value and altruism. Biol Theory 2: 143–155.

[16] Flinn MV, Alexander RD (2007) Runaway social selection. In: Gangestad SW, Simpson JA, editors. The evolution of mind. New York: Guilford Press. 249–255.

[17] West Eberhard MJ (1979) Sexual selection, social competition, and evolution. Proc Am Philos Soc 123: 222–234.

[18] West Eberhard MJ (1983) Sexual selection, social competition, and speciation. Q Rev Biol 58: 155–183.

[19] Barclay P, Willer R (2007) Partner choice creates competitive altruism in humans. Proc R Soc Lond B Biol Sci 274: 749–753. 10.1098/rspb.2006.0209 PMC2197220 17255001

[20] Chiang YS (2010) Self-interested partner selection can lead to the emergence of fairness. Evol Hum Behav 31: 265–270.

[21] Sylwester K, Roberts G (2010) Cooperators benefit through reputation-based partner choice in economic games. Biol Lett 6: 659–662. 20410026 10.1098/rsbl.2010.0209 PMC2936156

[22] Amundsen T (2000) Why are female birds ornamented? Trends Ecol Evol 15: 149–155. 10717684 10.1016/s0169-5347(99)01800-5

[23] Nesse RM (2010) Social selection and the origins of culture. In: Schaller M, Norenzayan A, Heine SJ, Yamagishi T, Kameda T, editors. Evolution, culture, and the human mind. New-York: Psychology Press.

[24] DuVal EH (2007) Cooperative display and lekking behavior of the lance-tailed manakin (Chiroxiphia lanceolata). Auk 124: 1168–1185.

[25] McDonald DB (1989) Cooperation under Sexual Selection - Age-Graded Changes in a Lekking Bird. Am Nat 134: 709–730.

[26] McDonald DB (1989) Correlates of Male Mating Success in a Lekking Bird with Male Male Cooperation. Anim Behav 37: 1007–1022.

[27] Duffy KG, Wrangham RW, Silk JB (2007) Male chimpanzees exchange political support for mating opportunities. Curr Biol 17: R586–R587. 17686425 10.1016/j.cub.2007.06.001

[28] Farrelly D, Lazarus J, Roberts G (2007) Altruists Attract. Evol Psychol 5: 313–329.

[29] Phillips T, Barnard C, Ferguson E, Reader T (2008) Do humans prefer altruistic mates ? Testing a link between sexual selection and altruism towards non-relatives. Br J Psychol 99: 555–572. 18817596 10.1348/000712608X298467

[30] Iredale W, Van Vugt M, Dunbar R (2008) Showing off in humans : male generosity as a mating signal. Evol Psychol 6: 386–392.

[31] Flinn MV, Ponzi D, Muehlenbein MP (2012) Hormonal Mechanisms for Regulation of Aggression in Human Coalitions. Hum Nat 23: 68–88. 22415579 10.1007/s12110-012-9135-y

[32] Roney JR, Mahler SV, Maestripieri D (2003) Behavioral and hormonal responses of men to brief interactions with women. Evol Hum Behav 24: 365–375.

[33] Ronay R, Von Hippel W (2010) The presence of an attractive woman elevates testosterone and physical risk taking in young men. Soc Psychol Personal Sci 1: 57–64.

[34] Gintis H, Smith EA, Bowles S (2001) Costly signaling and cooperation. J Theor Biol 213: 103–119. 11708857 10.1006/jtbi.2001.2406

[35] Zahavi A (1995) Altruism as a handicap - the limitations of kin selection and reciprocity. J Avian Biol 26: 1–3.

[36] Miller GF (2007) Sexual selection for moral virtues. Q Rev Biol 82: 97–125. 17583267 10.1086/517857

[37] Van Vugt M, Iredale W (2012) Men behaving nicely: Public goods as peacock tails. Br J Psychol. doi: 10.1111/j.2044-8295.2011.02093.x. 10.1111/j.2044-8295.2011.02093.x 23320439

[38] Smith EA, Bird RB, Bird DW (2003) The benefits of costly signaling: Meriam turtle hunters. Behav Ecol 14: 116–126.

[39] Smith EA, Bird RLB (2000) Turtle hunting and tombstone opening: public generosity as costly signaling. Evol Hum Behav 21: 245–261. 10899477 10.1016/s1090-5138(00)00031-3

[40] Henrich J (2000) Does culture matter in economic behavior? Ultimatum game bargaining among the Machiguenga of the Peruvian Amazon. Am Econ Rev 90: 973–979.

[41] Henrich J, Boyd R, Bowles S, Camerer C, Fehr E, et al (2005) “Economic man” in cross-cultural perspective: Behavioral experiments in 15 small-scale societies. Behav Brain Sci 28: 795–855. 16372952 10.1017/S0140525X05000142

[42] Rege M, Telle K (2004) The impact of social approval and framing on cooperation in public good situations. J Public Econ 88: 1625–1644.

[43] Charness G, Rustichini A (2011) Gender differences in cooperation with group membership. Games Econ Behav 72: 77–85.

[44] Greig F, Bohnet I (2009) Exploring gendered behavior in the field with experiments: Why public goods are provided by women in a Nairobi slum. J Econ Behav Organ 70: 1–9.

[45] Lamba S, Mace R (2011) Demography and ecology drive variation in cooperation across human populations. Proc Natl Acad Sci U S A 108: 14426–14430. 21831836 10.1073/pnas.1105186108 PMC3167540

[46] Benz M, Meier S (2008) Do people behave in experiments as in the field? evidence from donations. Experimental Economics 11: 268–281.

[47] Fehr E, Leibbrandt A (2011) A field study on cooperativeness and impatience in the Tragedy of the Commons. J Public Econ 95: 1144–1155.

[48] Rustagi D, Engel S, Kosfeld M (2010) Conditional Cooperation and Costly Monitoring Explain Success in Forest Commons Management. Science 330: 961–965. 21071668 10.1126/science.1193649

[49] Clutton Brock TH (1989) Mammalian mating systems. Proc R Soc Lond B Biol Sci 236: 339–372. 2567517 10.1098/rspb.1989.0027

[50] Geary DC (2000) Evolution and proximate expression of human paternal investment. Psychol Bull 126: 55–77. 10668350 10.1037/0033-2909.126.1.55

[51] Courtiol A, Raymond M, Faurie C (2009) Birth order affects behaviour in the investment game: firstborns are less trustful and reciprocate less. Anim Behav 78: 1405–1411.

[52] Whittingham MJ, Stephens PA, Bradbury RB, Freckleton RP (2006) Why do we still use stepwise modelling in ecology and behaviour? J Anim Ecol 75: 1182–1189. 16922854 10.1111/j.1365-2656.2006.01141.x

[53] R Development Core Team (2010) R: a language and environment for statistical computing. Vienna, Austria: R Foundation for Statistical Computing.

[54] Chaudhuri A (2011) Sustaining cooperation in laboratory public goods experiments: a selective survey of the literature. Experimental Economics 14: 47–83.

[55] Fischbacher U, Gachter S (2010) Social Preferences, Beliefs, and the Dynamics of Free Riding in Public Goods Experiments. Am Econ Rev 100: 541–556.

[56] Keser C (1996) Voluntary contributions to a public good when partial contribution is a dominant strategy. Econ Lett 50: 359–366.

[57] Keser C, Van Winden F (2000) Conditional cooperation and voluntary contributions to public goods. Scand J Econ 102: 23–39.

[58] Llaurens V, Raymond M, Faurie C (2009) Ritual fights and male reproductive success in a human population. J Evol Biol 22: 1854–1859. 19583698 10.1111/j.1420-9101.2009.01793.x

[59] Bowles S (2006) Group competition, reproductive leveling, and the evolution of human altruism. Science 314: 1569–1572. 17158320 10.1126/science.1134829

