# Supplementary material for: Sexual Selection of Human Cooperative Behaviour: An Experimental Study in Rural Senegal
Source: PLoS One. 2012 Sep 12;7(9):e44403. doi: 10.1371/journal.pone.0044403 (PMC3440379; doi:10.1371/journal.pone.0044403)
Supplement: Table S1 — Tobit regression models of (1) men’s and (2) women’s contributions to the public good in the first period of the PGG. For each variable, the estimate, standard error of the mean (SE), χ2 statistic, degrees of freedom (df), and p-value of the χ2 test are given. For categorical variables, the estimates are for one category compared to the reference category (underlined term). The results of the models controlling for age instead of the number of offspring were not qualitatively different (available upon request). (PDF) [file pone.0044403.s001.pdf]

Table S1. Tobit regression models of (1) men's and (2) women's contributions to the public good in the first period of the PGG. For each variable, the estimate, standard error of the mean (SE), χ^2 statistic, degrees of freedom (df), and *p*-value of the χ^2 test are given. For categorical variables, the estimates are for one category compared to the reference category (underlined term). The results of the models controlling for age instead of the number of offspring were not qualitatively different (available upon request).

1. Men (*n* = 136)

Predictor variables		Estimate	(SE)	χ2	df	*p* value
(Intercept)		120.5	21.0	33	1	<0.0001
Category of observers				3.2	2	0.21
	Young women / Men	9.9	10.7			
	Old women / Men	20.2	11.4			
Villages				43.8	4	<0.0001
	Village A / B	-45.1	17.5			
	Village C / B	-1.4	18.6			
	Village D / B	-74.6	18.1			
	Village E / B	-6.8	17.2			
SES		-4.1	4.4	0.9	1	0.36
Number of offspring		-0.7	1.3	0.3	1	0.60
Birth order	Other children / Firstborn	4.5	10.8	0.2	1	0.67
Wald χ2				56.1	9.00	<0.0001
Log-likelihood		-734.7				

2. Women (*n* = 90)

Predictor variables		Estimate	SE	χ2	df	*p* value
(Intercept)		136.4	32.0	18.2	1	<0.0001
Category of observers	Young women / Men	34.9	13.0	7.2	1	0.007
Villages				15.8	4	0.003
	Village A / B	-82.6	31.0			
	Village C / B	-84.6	31.9			
	Village D / B	-111.4	30.5			
	Village E / B	-69.8	31.6			
SES		-1.3	5.8	0.05	1	0.82
Number of offspring		6.0	2.2	7.4	1	0.006
Birth order	Other children / Firstborn	7.6	14.6	0.3	1	0.60
Wald χ2				36.7	8	<0.0001
Log-likelihood		-498.2				
