# Supplementary material for: Sexual Selection of Human Cooperative Behaviour: An Experimental Study in Rural Senegal
Source: PLoS One. 2012 Sep 12;7(9):e44403. doi: 10.1371/journal.pone.0044403 (PMC3440379; doi:10.1371/journal.pone.0044403)
Supplement: Table S2 — Random Tobit regression models of (1) men’s and (2) women’s contributions to the public good in periods 2 through 5 of the PGG. For each variable, the estimate, standard error of the mean (SE), χ2 statistic, degrees of freedom (df), and p-value of the χ2 test are given. For categorical variables, the estimates are for one category compared to the reference category (underlined term). The results of the models controlling for age instead of the number of offspring were not qualitatively different (available upon request). (PDF) [file pone.0044403.s002.pdf]

Table S2. Random Tobit regression models of (1) men's and (2) women's contributions to the public good in periods 2 through 5 of the PGG. For each variable, the estimate, standard error of the mean (SE), χ^2 statistic, degrees of freedom (df), and *p*-value of the χ^2 test are given. For categorical variables, the estimates are for one category compared to the reference category (underlined term). The results of the models controlling for age instead of the number of offspring were not qualitatively different (available upon request).

1. Men (*n* =541)

Predictor variables		Estimate	(SE)	$\chi 2$	df	*p* value
(Intercept)		129.5	(17.4)	55.5	1	<0.0001
Category of observers				5.1	2	0.078
	Young women / Men	8.1	(7.0)			
	Old women / Men	19.8	(8.8)			
Villages				66.1	4	<0.0001
	Village A / B	-34.0	(14.8)			
	Village C / B	6.4	(14.5)			
	Village D / B	-64.5	(15.1)			
	Village E / B	-0.9	(14.4)			
SES		0.4	(3.1)	0.02	1	0.9
Number of offspring		-1.3	(0.9)	2.2	1	0.14
Birth order	Other children / Firstborn	2.9	(7.0)	0.2	1	0.68
Group contribution t-1		-0.01	(0.01)	1.9	1	0.17
Log-likelihood		-2839.9				

2. Women (*n* = 374)

Predictor variables		Estimate	(SE)	$\chi 2$	df	*p* value
(Intercept)		79.6	(19.5)	16.6	1	<0.0001
Category of observers	Young women / Men	17.4	(5.7)	9.5	1	0.002
Villages				27.3	4	<0.0001
	Village A / B	-3.2	(14.9)			
	Village C / B	-11.9	(16.2)			
	Village D / B	-47.3	(16.2)			
	Village E / B	-19.4	(14.7)			
SES		0.4	(2.6)	0.02	1	0.88
Number of offspring		3.1	(1.2)	6.9	1	0.009
Birth order	Other children / Firstborn	9.1	(9.8)	0.9	1	0.36
Group contribution t-1		0.03	(0.01)	5.5	1	0.02
Log-likelihood		-1973.4				
