# Supplementary material for: Sexual Selection of Human Cooperative Behaviour: An Experimental Study in Rural Senegal
Source: PLoS One. 2012 Sep 12;7(9):e44403. doi: 10.1371/journal.pone.0044403 (PMC3440379; doi:10.1371/journal.pone.0044403)
Supplement: Table S3 — GLMMs of (1) men’s and (2) women’s probability of donating to the school canteen. For each variable, the estimate, standard error of the mean (SE), χ2 statistic, degrees of freedom (df), and p-value of the likelihood ratio test of the comparison between the full model and the model without the factor, are given. For categorical variables, the estimates are for one category compared to the reference category (underlined term). The results of the models controlling for age instead of the number of offspring were not qualitatively different (available upon request). (PDF) [file pone.0044403.s003.pdf]

Table S3. GLMMs of (1) men's and (2) women's probability of donating to the school canteen. For each variable, the estimate, standard error of the mean (SE), χ^2 statistic, degrees of freedom (df), and *p*-value of the likelihood ratio test of the comparison between the full model and the model without the factor, are given. For categorical variables, the estimates are for one category compared to the reference category (underlined term). The results of the models controlling for age instead of the number of offspring were not qualitatively different (available upon request).

1. Men (*n* = 138)

Predictor variables		Estimate	SE	χ2	df	*p* value
(Intercept)		1.1	1.3			
Category of observers				12.9	2	0.002
	Young women / Men	1.2	0.6			
	Old women / Men	2.5	0.7			
Villages				18.0	4	0.001
	Village A / B	-1.6	1.2			
	Village C / B	-3.2	1.2			
	Village D / B	0.01	1.3			
	Village E / B	-0.5	1.2			
SES		0.4	0.3	2.8	1	0.1
Number of offspring		0.002	0.1	<0.0001	1	1.0
Birth order	Other children / Firstborn	-0.01	0.6	<0.0001	1	1.0
Payoff		0.2	0.3	0.5	1	0.5
Random term		Variance	SE			
ID groups		<0.0001	<0.0001	14.1	1	0.0002

2. Women (*n* = 98)

Predictor variables		Estimate	SE	χ2	df	*p* value
(Intercept)		18.1	2797.3			
Category of observers	Young women / Men	1.2	0.6	4.5	1	0.03
Villages				27.7	4	<0.0001
	Village A / B	-18.8	2797.3			
	Village C / B	-20.1	2797.3			
	Village D / B	-16.7	2797.3			
	Village E / B	-16.5	2797.3			
SES		0.3	0.3	0.8	1	0.4
Number of offspring		-0.02	0.1	0.1	1	0.8
Birth order	Other children / Firstborn	-1.0	0.7	1.9	1	0.2
Payoff		-0.2	0.3	0.3	1	0.6
Random term		Variance	SE			
ID groups		<0.0001	<0.0001	10.7	1	0.001
