# Supplementary material for: Sexual Selection of Human Cooperative Behaviour: An Experimental Study in Rural Senegal
Source: PLoS One. 2012 Sep 12;7(9):e44403. doi: 10.1371/journal.pone.0044403 (PMC3440379; doi:10.1371/journal.pone.0044403)
Supplement: Table S4 — LMMs of the amounts donated by (1) men and (2) women to the school canteen for individuals who made a non-zero donation. For each variable, the estimate, standard error of the mean (SE), F statistic, numerator degrees of freedom (df), denominator degrees of freedom (dfden), and p-value of the likelihood ratio test of the comparison between the full model and the model without the factor, are given. For categorical variables, the estimates are for one category compared to the reference category (underlined term). The results of the models controlling for age instead of number of offspring were not qualitatively different (available upon request). (PDF) [file pone.0044403.s004.pdf]

Table S4. LMMs of the amounts donated by (1) men and (2) women to the school canteen for individuals who made a non-zero donation. For each variable, the estimate, standard error of the mean (SE), *F* statistic, numerator degrees of freedom (df), denominator degrees of freedom (dfden), and *p*-value of the likelihood ratio test of the comparison between the full model and the model without the factor, are given. For categorical variables, the estimates are for one category compared to the reference category (underlined term). The results of the models controlling for age instead of number of offspring were not qualitatively different (available upon request).

1. Men (n = 98)

Predictor variables		Estimate	SE	*F* value	df	dfden	*p* value
(Intercept)		5.2	0.4	245.7	1	60	<0.0001
Category of observers				4.5	2	27	0.02
	Young women / Men	0.03	0.2			27	
	Old women / Men	-0.5	0.2			27	
Villages				1.7	4	27	0.18
	Village A / B	-0.1	0.3		1	27	
	Village C / B	-0.6	0.4		1	27	
	Village D / B	-0.5	0.3		1	27	
	Village E / B	-0.5	0.3		1	27	
SES		0.02	0.1	0.1	1	60	0.74
Number of offspring		0.02	0.02	0.6	1	60	0.43
Birth order	Other children / Firstborn	0.2	0.1	1.9	1	60	0.17
Payoff		0.01	0.1	0.0	1	60	0.85
Random term					$\chi2$	df	*p* value
ID groups					17.6	1	<0.0001

2. Women (n = 52)

Predictor variables		Estimate	SE	*F* value	*df*	*dfden*	*p* value
(Intercept)		96.4	92.2	1.1	1	27	0.31
Category of observers	Young women / Men	-32.2	33.6	0.9	1	15	0.35
Villages				4.2	4	15	0.02
	Village A / B	136.7	84.8			15	
	Village C / B	126.5	104.9			15	
	Village D / B	23.2	84.7			15	
	Village E / B	168.4	78.6			15	
SES		8.0	12.7	0.4	1	27	0.53
Number of offspring		13.7	5.5	6.1	1	27	0.02
Birth order	Other children / Firstborn	-26.8	34.7	0.6	1	27	0.45
Payoff		-24.8	17.5	2.0	1	27	0.17
Random term					$\chi2$	df	*p* value
ID groups					2.2	1	0.1
