# Supplementary material for: Sexual Selection of Human Cooperative Behaviour: An Experimental Study in Rural Senegal
Source: PLoS One. 2012 Sep 12;7(9):e44403. doi: 10.1371/journal.pone.0044403 (PMC3440379; doi:10.1371/journal.pone.0044403)
Supplement: Table S5 — General linear regression of the change in men’s attractiveness as rated by (1) young female and (2) old female observers as a function of the men’s (a) contributions to the public good and (b) generosity towards children. For each factor, the estimate, standard error of the mean (SE), degrees of freedom (df), F statistic, and p-value of the likelihood ratio test of the comparison between the full model and the model without the factor, are given. For categorical variables, the estimates are for one category compared to the reference category (underlined term). (PDF) [file pone.0044403.s005.pdf]

Table S5. General linear regression of the change in men's attractiveness as rated by (1) young female and (2) old female observers as a function of the men's (a) contributions to the public good and (b) generosity towards children. For each factor, the estimate, standard error of the mean (SE), degrees of freedom (df), *F* statistic, and *p*-value of the likelihood ratio test of the comparison between the full model and the model without the factor, are given. For categorical variables, the estimates are for one category compared to the reference category (underlined term).

1. Men as rated by young female observers

Response variable				Estimate	SE	*F value*	df	*p* value
Change in perceived attractiveness	(a) (*n* = 54)	Intercept		2.0	0.6			
		Contribution to the public		-0.0001	0.003	0.001	1	0.97
		Attractiveness before		-0.6	0.1	21.6	1	<0.0001
		Village				0.3	4	0.86
			Village A / B	0.01	0.3			
			Village C / B	-0.01	0.3			
			Village D / B	-0.0003	0.3			
			Village E / B	-0.2	0.3			
		Age		-0.01	0.01	7.3	1	0.01
		SES		0.3	0.1	13.5	1	<0.01
	(b) (*n* = 57)	Intercept		2.0	0.5			
		Donation towards children		-0.0002	0.001	0.1	1	0.73
		Attractiveness before		-0.6	0.1	20.1	1	<0.0001
		Village				0.7	4	0.60
			Village A / B	-0.1	0.3			
			Village C / B	-0.3	0.4			
			Village D / B	0.2	0.4			
			Village E / B	-0.2	0.3			
		Age		-0.01	0.01	4.9	1	0.03
		SES		0.3	0.1	10.6	1	<0.01
		Payoff of the PGG		0.2	0.1	2.2	1	0.14

2. Men as rated by old female observers

Response variable				Estimate	SE	*F* value	df	*p* value
Change in perceived attractiveness	(a) (*n* = 39)	Intercept		1.0	1.0			
		Contribution to the public		0.01	0.005	3.0	1	0.09
		Attractiveness before		-0.7	0.2	17.3	1	<0.001
		Village				2.2	4	0.09
			Village A / B	-0.1	0.5			
			Village C / B	-0.6	0.6			
			Village D / B	1.3	0.7			
			Village E / B	0.1	0.4			
		Age		0.002	0.01	0.05	1	0.83
		SES		0.1	0.2	0.3	1	0.56
	(b) (*n* = 36)	Intercept		2.2	0.8			
		Donation towards children		0.003	0.002	1.7	1	0.21
		Attractiveness before		-0.6	0.2	10.1	1	<0.01
		Village				1.2	4	0.32
			Village A / B	-0.7	0.5			
			Village C / B	-0.7	0.6			
			Village D / B	0.1	1.0			
			Village E / B	0.002	0.5			
		Age		-0.004	0.01	0.1	1	0.72
		SES		0.1	0.2	0.2	1	0.67
		Payoff of the PGG		-0.3	0.2	1.8	1	0.19
