# Supplementary material for: Sexual Selection of Human Cooperative Behaviour: An Experimental Study in Rural Senegal
Source: PLoS One. 2012 Sep 12;7(9):e44403. doi: 10.1371/journal.pone.0044403 (PMC3440379; doi:10.1371/journal.pone.0044403)
Supplement: Table S6 — General linear regression of the change in women’s attractiveness as rated by male observers as a function of the women’s (a) contributions to the public good and (b) generosity towards children. For each factor, the estimate, standard error of the mean (SE), degrees of freedom (df), F statistic and p-value of the likelihood ratio test of the comparison between the full model and the model without the factor, are given. For categorical variables, the estimates are for one category compared to the reference category (underlined term). (PDF) [file pone.0044403.s006.pdf]

Table S6. General linear regression of the change in women's attractiveness as rated by male observers as a function of the women's (a) contributions to the public good and (b) generosity towards children. For each factor, the estimate, standard error of the mean (SE), degrees of freedom (df), *F* statistic and *p*-value of the likelihood ratio test of the comparison between the full model and the model without the factor, are given. For categorical variables, the estimates are for one category compared to the reference category (underlined term).

Response variable				Estimate	SE	*F* value	df	*p* value
Change in perceived attractiveness	(a) (*n* = 38)	Intercept		1.8	0.7			
		Contribution to the public good		0.003	0.003	0.7	1	0.40
		Attractiveness before		-0.6	0.1	25.6	1	<0.0001
		Villages				0.3	4	0.26
			Village A / B	0.4	0.3			
			Village C / B	-0.1	0.4			
			Village D / B	0.3	0.4			
			Village E / B	-0.3	0.3			
		Age		-0.02	0.01	0.1	1	0.06
		SES		-0.03	0.1	0.8	1	0.77
	(b) (*n* = 43)	Intercept		1.6	0.7			
		Donation towards children		<0.001	0.001	0.5	1	0.49
		Attractiveness before		-0.5	0.1	19.7	1	<0.0001
		Villages				1.3	4	0.29
			Village A / B	0.4	0.4			
			Village C / B	-0.05	0.4			
			Village D / B	0.3	0.5			
			Village E / B	-0.1	0.4			
		Age		-0.01	0.01	1.9	1	0.18
		SES		0.05	0.1	0.3	1	0.61
		Payoff of the PGG		0.04	0.1	0.1	1	0.74
